# Restoration of Deafferentation Reduces Tinnitus, Anxiety, and Depression: A Retrospective Study on Cochlear Implant Patients

**DOI:** 10.1155/2021/6678863

**Published:** 2021-06-24

**Authors:** Juanmei Yang, Jing Song, Xiang Zhao, Carol Pang, Ning Cong, Zhao Han

**Affiliations:** ^1^Department of Otology and Skull Base Surgery, Eye Ear Nose and Throat Hospital, Fudan University, Shanghai 200031, China; ^2^Shanghai Clinical Medical Center of Hearing Medicine, Shanghai 200031, China; ^3^Key Laboratory of Hearing Medicine of National Health Commission of the People's Republic of China, Shanghai 20031, China; ^4^Research Institute of Otolaryngology, Fudan University, Shanghai 200031, China; ^5^Department of Traditional Chinese Medicine, Shandong Zaozhuang Municipal Hospital, Shandong Zaozhuang 277100, China; ^6^Audiology, Speech & Medical Research Institute, Nurotron Biotechnology Co., Ltd, Hangzhou 310011, China; ^7^Department of Otorhinolaryngology, Huadong Hospital Affiliated with Fudan University, 200040, No. 221 West Yan'an Road, Jing An District, Shanghai, China

## Abstract

Patients with profound bilateral deafness (BD) are prone to suffering from tinnitus, which further leads to psychological comorbidities and makes it more difficult for patients to communicate with people. This study was aimed at investigating the effect of cochlear implants (CIs) on tinnitus distress and psychological comorbidities in patients with profound BD. This multicenter retrospective study reviewed 51 patients with severe postlingual BD who underwent cochlear implantation; 49 patients underwent unilateral cochlear implantation, and 2 patients underwent bilateral cochlear implantation. The patients were asked to complete all the questionnaires, including the tinnitus handicap inventory (THI), the visual analog scale (VAS) score, the Hospital Anxiety and Depression Scale Questionnaire (HADS), the Categories of Auditory Performance (CAP), and the Speech Intelligibility Rating (SIR), at least 4 months after implantation when the CI was on or off, in approximately May-June 2019. In our study, 94% (48/51) of BD patients suffered from tinnitus before CI, and 77% (37/48) of them suffered from bilateral tinnitus. In addition, 50.9% (26/51) of the CI patients were suffering from anxiety, 52.9% (27/51) of them were suffering from depression (score ≥ 8), and 66.7% (34/51) (27/51) of them were suffering from anxiety or depression. Cochlear implantation could reduce tinnitus more obviously when the CI was on than when the CI was off. Cochlear implantation also reduced anxiety/depression severity. There were significantly positive correlations between tinnitus severity and anxiety/depression severity before and after surgery. Moreover, hearing improvement is positively correlated with reduction level of tinnitus, the better hearing, and the lesser severity of tinnitus. Thus, along with effective restoration of deafferentation, cochlear implantation shows positive therapeutic effects on tinnitus and psychological comorbidities, providing a reference for future clinical and research work.

## 1. Introduction

Tinnitus, which literally means “ringing in the ears,” is defined by the perception of sound or noise in the absence of an external physical sound source. The prevalence of tinnitus in adults is 10-15%. In the affected subgroup of patients, it causes extreme distress with far-reaching consequences for daily activities and quality of life [1]. In addition, tinnitus can cause an overall perceived handicap that can include hearing difficulties, anxiety, depression, inability to relax, and sleep difficulties [2]. In sensorineural hearing-impaired patients, tinnitus has a higher prevalence, but this association is not simple or straightforward because some people with troublesome tinnitus have audiometrically normal hearing; conversely, many people with hearing loss do not report tinnitus [1, 3].

Cochlear damage is a trigger factor for tinnitus. Tinnitus is generated by a series of changes in central auditory pathways, such as the cochlear nucleus (CN), inferior colliculus (IC), the medial geniculate body (MGB), and auditory cortex (AC) to compensate for the loss of this input when the electrical input of the cochlea decreases or disappears [4]. Based on the tinnitus mechanism by which injury to the peripheral auditory system induces plasticity in the auditory central system, many treatment algorithms, including sound therapy, try to recorrect plasticity and restore hearing loss with hearing aids to reduce tinnitus [1, 5]. Cochlear implantation involves the surgical placement of an electrode array within the cochlea to stimulate spiral ganglion cells electrically to convey auditory information [6]. Recently, cochlear implants have been used more frequently to restore deafferentation in profound single-side or bilateral postlingual deafness patients [7–11]. The prevalence rates of tinnitus in postlingual profound BD patients in previous studies are different, ranging from 67% to 86% in cochlear implant (CI) candidates ([7, 10–14]). Although many reports have explored the relationships between CI and tinnitus/depression/anxiety, it is still controversial whether CI may induce or reduce tinnitus distress [11, 12, 15]. This study was aimed at investigating the influence of restoring deafferentation with cochlear implants (CIs) on tinnitus distress and psychological comorbidities in profound BD patients. Fifty-one CI participants with postlingually acquired profound BD were involved in this retrospective study. We investigated the severity of tinnitus, anxiety, and depression before and after CI surgery according to different questionnaires to explore the response of postlingually profound BD patients who receive cochlear implants which will provide clues regarding the tinnitus mechanism and provide directions for the treatment of tinnitus.

## 2. Patients and Methods

This study was performed at multiple centers over a period of approximately 6 years (2013.9.16-2019.6.6). Fifty-one adult patients with acquired bilateral profound deafness were enrolled in the study, which was approved by the local ethics committee. The main inclusion criteria were adult CI patients with profound bilateral hearing loss, intact auditory nerves, and no obvious malformation of the cochlea.

The patients were asked to complete all the questionnaires at least 4 months after implantation. There were 24 males and 27 females. The mean age at the time of implantation was 41.0 ± 17.0 years (range 19.0–74.0 years). The mean duration of deafness before implantation was 8.0 ± 7.2 years (range 0.5–27 years). The mean time post-CI at the moment of completing the questionnaire was 18.0 ± 16.7 months (range 3–69 months). The cochlear type for every patient was the Nurotron (CS-10A). Two of the patients (2/51) received bilateral CIs (49/51), and the others received unilateral CIs. A postoperative X-ray was taken to verify successful intracochlear electrode insertion for each patient. Thirty-two patients had worn hearing aids before CI (15 bilaterally and 17 unilaterally). Nineteen patients had not worn hearing aids before CI. Student's *t*-test and ANOVA were used to assess VAS, THI, HAD, CAP, and SIR scores before and after CI. Statistical significance was defined as ^∗^*p* < 0.05 and ^∗∗^*p* < 0.01. The relationship between hearing ability and scores from the questionnaires was tested using Spearman's correlation test.

### 2.1. Tinnitus Handicap Inventory (THI) and Visual Analog Scale (VAS) Score

The THI is an internationally validated tinnitus scoring questionnaire developed by Newman et al. in 1996 [16]. It consists of 25 questions with the purpose of evaluating the functional, emotional, and catastrophic effects of tinnitus. The three choices for each question are “Yes,” “Occasionally,” and “No,” and scores of 4, 2, and 0 are applied to the choices. The CI recipients were required to select one choice among the three recipients. They were also asked to complete the THI to assess their tinnitus impact on their psychology and activities of daily living. There were five tinnitus severity levels determined by the total score: slight (0–16), mild (18–36), moderate (38–56), severe (58–76), or catastrophic (78–100).

The VAS was used to evaluate the severity of tinnitus at the same time as the THI was assessed [17]. The patients were asked to complete both questionnaires with the CI device on and off. Patients were asked to mark a single point between 0 and 10 to indicate their feelings. For example, the question of satisfaction for CI sounds was evaluated for whether tinnitus interfered with the listening effect of the CI. A score of 10 on the scale was identified as “too noisy and annoying,” and a score of 0 suggested “absolutely clear and satisfying.”

### 2.2. Hospital Anxiety and Depression Scale Questionnaire (HADS)

A hearing handicap and chronic tinnitus can be associated with depressive symptoms such as anxiety or emotions such as helplessness. We used the translated and validated Dutch version of the Hospital Anxiety and Depression Scale (HADS). The HADS questionnaire assessed the presence and severity of mild and even subsyndrome degrees of anxiety and depression [18, 19]. The questionnaire contains 14 items, i.e., 7 about anxiety and 7 about depression. Patients answered the questions on a scale of 0–3. Subscale scores can be calculated for anxiety and depression. Scores below 7 indicate neither an anxiety nor a depression problem, scores between 8 and 10 suggest a potential anxiety or depression disorder, and scores beyond 11 indicate definite cases of anxiety or depression. The scores of the two subscales of anxiety and depression were as follows: 0-7 was negative; 8-10 was mild; 11-14 was classified as moderate; and 15-21 was classified as severe. Studies have found that the HADS has good reliability and validity. Taking 9 points as the critical value of anxiety and depression yielded good sensitivity and specificity. Therefore, the use of this critical point is recommended.

### 2.3. Categories of Auditory Performance (CAP) and Speech Intelligibility Rating (SIR)

Categories of Auditory Performance (CAP) and the Speech Intelligibility Rating (SIR) were developed by the University of Nottingham for the assessment of children's daily auditory and speech ability [20] and have been widely used in the assessment of the effect of speech rehabilitation after cochlear implantation in young children [21]. In this study, we used this tool to evaluate adult patients. For each patient, the measures were assessed at least 4 months after CI surgery. The higher the score, the better the auditory comprehension and the better the speech recognition.

### 2.4. Statistical Analyses

All data were analyzed using SPSS 18.0 (SPSS Inc., Chicago, IL, USA). Normal distribution of the data was verified with the Kolmogorov–Smirnov test. Student's *t*-test and ANOVA were used to assess the VAS, THI, HAD, CAP, and SIR scores before and after CI with the device on and off. Statistical significance was defined as ^∗^*p* < 0.05 and ^∗∗^*p* < 0.01. The relationships between hearing ability and scores from the questionnaires were tested using Spearman's correlation test.

## 3. Results

### 3.1. Characteristics of the CI Patients

**Figure 1:**
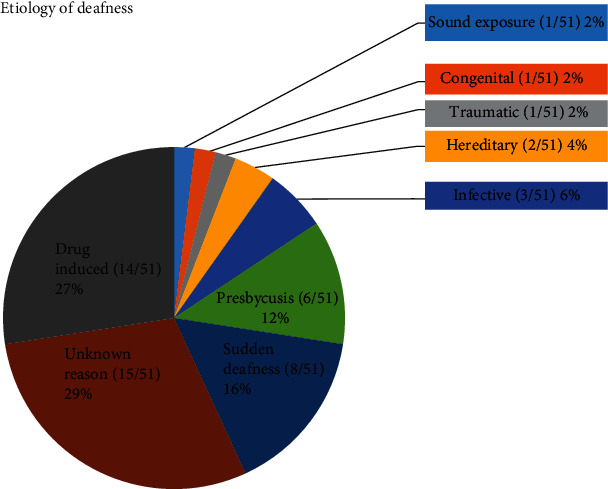
Etiology of deafness of CI candidates.

Fifty-one postlingual profound BD patients (27 women, 24 men) in this study were implanted with a multichannel cochlear implant manufactured by Nurotron. Surgeries were performed between 2013 and 2019. The mean age at the time of implantation was 41.0 ± 17.0 years old (range 19.0-74.0 years old). The mean duration of deafness before implantation was 8.0 ± 7.2 years (range 0.5-27 years). The mean time post-CI at the moment of completing the questionnaire was 18.0 ± 16.7 months (range 3-69 months). The other preoperative characteristics are shown in Table 1 and Figure 1.

### 3.2. Most of the Profound BD CI Candidates with Tinnitus Also Suffered from Anxiety and Depression Distress before CI

According to our results, 94% (48/51) of the CI patients suffered from tinnitus before CI surgery, and 77.1% (37/48) of them had bilateral tinnitus. The average THI scores were 48.3 ± 22.2 in males and 57.3 ± 29.9 in females, and there was no significant difference between the male and female patients (*p* = 0.24). The THI scores showed that 2% of them (1/48) were suffering from only slight tinnitus (0-16), 25.5% (14/48) were suffering from mild tinnitus (18-36), 19.6% (10/48) were suffering from moderate tinnitus (38-56), 23.5% (12/48) were suffering from severe tinnitus (58-76), and 21.6% (11/48) were suffering from catastrophic tinnitus (78-100). The proportion of CI patients with severe and catastrophic tinnitus before CI surgery was 45.1%. The etiology of deafness of CI candidates includes drug induced (27%, 14/51), sudden hearing loss (16%, 8/51), presbycusis (6/51, 12%), infective (3/51, 6%), hereditary (2/51, 4%), traumatic (1/51, 2%), congenital (1/51, 2%), sound exposure (1/51, 2%), and unknown reason (15/51, 29%) shown in Figure 1.

In addition, according to our results, 50.9% (26/51) of the CI patients suffered from anxiety before CI surgery (≥8), 84.6% (22/26) were at a mild level (8-10), and 15.4% (4/26) were at a moderate level (11-14). Overall, 52.9% (27/51) of the CI patients were suffering from depression before CI surgery, with 51.9% (14/27) of them exhibiting mild depression (8-10), 44.4% (12/27) moderate depression (11-14), and 3.4% (1/27) severe depression (15-21); 37.2% (19/51) of CI candidates experienced both anxiety and depression. The detailed characteristics are shown in Table 1.

### 3.3. The Relationships between Depression/Anxiety Levels and Tinnitus Severity Levels

Pearson's relative statistic was used to test the relationships between tinnitus severity levels and depression/anxiety levels. The results showed that the THI score was positively correlated with depression and anxiety scores before CI surgery (*p* < 0.01, *n* = 51, bilateral), and the THI score was positively correlated with depression scores after CI surgery (*p* < 0.01, *n* = 51, bilateral). However, there was no correlation between the THI score and the anxiety score after CI surgery (*p* = 0.06) (Table 2).

### 3.4. Tinnitus, Depression, and Anxiety Could Be Suppressed in Profound BD Patients after CI

The tinnitus prevalence rates of CI patients were 94% (48/51) before CI and 98% (50/51) after CI when the device is off and 94% (48/51) when the device is on. It is worth noting that tinnitus disappeared after CI in 3 patients when the CI device is on (CI on) and in 1 patient when the CI device is off (CI off). We also found that 3 patients without tinnitus before surgery suffered from tinnitus after CI.

The preoperative average THI score was 53.1 ± 27.5, and the postoperative average THI scores were 43.2 ± 26.2 (CI off) and 29.1 ± 21.3 (CI on). For the average THI score, there was no significant difference between preoperative and CI-off scores (*p* = 0.06), but there was a significant difference between preoperative and CI-on scores (*p* < 0.01). Additionally, there was a significant difference between CI-on and CI-off scores (*p* < 0.01). In other words, tinnitus improvements of all CI patients were more obvious when the CI was on than off (see Table 3).

To obtain more details, further comparisons among patients were conducted according to different levels of tinnitus. For patients with catastrophic tinnitus, the average THI score decreased from 90.4 ± 7.1 preoperatively to 66.7 ± 31.4 with the CI off and 39.8 ± 32.2 with the CI on. There was a significant difference between the THI score of preoperative and CI-on conditions (*p* < 0.01) and between the preoperative and CI-off conditions (*p* = 0.02). There was no significant difference between CI-on and CI-off conditions (*p* = 0.06) (see Table 3).

For the patients with severe tinnitus, the average THI score decreased from 67.7 ± 6.1 preoperatively to 54.8 ± 21.1 postoperatively with the CI off and 35.8 ± 20.1 with the CI on. There was a significant difference between the THI score of preoperative and CI-on conditions (*p* < 0.01), and there was a significant difference between the CI-on and CI-off conditions (*p* = 0.03). However, there was no significant difference between the preoperative and postoperative scores of CI-off condition (*p* = 0.06) (see Table 3).

For the patients with moderate tinnitus, the average THI score decreased from 48.4 ± 6.1 preoperatively to 42.4 ± 8.1 postoperatively with the CI off and 32.6 ± 12.9 with the CI on. For the THI score, there was a significant difference between the preoperative and CI-on conditions (*p* < 0.01), there was no significant difference between the CI-on and CI-off conditions (*p* = 0.06), and there was no significant difference between the preoperative and postoperative CI groups when the CI was off (*p* = 0.08) (see Table 3).

For the patients with mild tinnitus, the average THI score decreased from 29.0 ± 5.0 preoperatively to 18.6 ± 9.1 postoperatively when the CI was off; when the CI was on, the average THI score was 13.8 ± 8.4. For the THI score, there was a significant difference between the preoperative and CI-on conditions (*p* < 0.01), there was no significant difference between the CI-on and CI-off conditions (*p* = 0.1), and there was a significant difference between the preoperative and postoperative CI groups when the CI was off (*p* < 0.01) (see Table 3).

For patients with slight tinnitus or no tinnitus (4/48) before surgery, the THI score increased from 12 preoperatively to 20 postoperatively when the CI was off and to 28 when the CI was on in one of the patients; for the other 3 patients without tinnitus before CI, the THI score increased from 0 to 12, 38, and 58, respectively, when the CI was off and to 12, 26, and 38, respectively, when the CI was on (see Table 3).

Although the prevalence rate of tinnitus is similar before and after CI, the tinnitus severity level decreased significantly after CI. The proportion of catastrophic tinnitus in the patients was reduced from 22.9% (11/48) preoperatively to 12% (6/50) when CI device was off and to 4.2% (2/48) when CI device was on. The proportion of severe tinnitus in the patients was reduced from 25% (12/48) preoperatively to 24% (12/50) when CI device was off and to 12% (5/48) when CI device was on. The proportion of moderate tinnitus in the patients was 20.8% (10/48) preoperatively and to 20% (10/50) when CI device was off and to 14.6% (7/48) when CI device was on. The proportion of mild tinnitus in the patients was 29.2% (14/48) preoperatively and to 22% (11/50) when CI device was off and to 35.4% (17/48) when CI device was on. The proportion of slight tinnitus in the patients was 2% (1/48) preoperatively and to 22% (11/50) when CI device was off and to 33.3% (16/48) when CI device was on.

According to the VAS results, the preoperative average VAS score was 3.9 ± 3.1, and the postoperative average VAS scores were 2.6 ± 2.8 when the CI device was off and 1.7 ± 2.2 when the CI device was on. There was no significant difference between the CI-off group and the preoperative group (*p* = 0.06). Meanwhile, there was a significant difference between the CI-on group and the preoperative group (*p* < 0.01), and there was a significant difference between the CI-on group and the CI-off group (*p* = 0.02) (see Table 3).

### 3.5. CI Could Reduce Anxiety and Depression Levels in Profound BD Patients

Before CI surgery, 50.9% (26/48) of the CI patients suffered from anxiety, and 52.9% (27/48) of them suffered from depression. After CI surgery, 73.1% (19/26) of patients who suffered from anxiety preoperatively reported anxiety scores within negative (no anxiety) range (*A* < 8), and 63% (17/27) of patients reported depression scores within negative (no depression) range (*D* < 8). According to our HADS results, the preoperative average anxiety score was 7.2 ± 2.5, and the postoperative average anxiety scores were 5.6 ± 2.6 when the CI device was off and 4.5 ± 2.4 when the CI device was on. There were significant differences between the CI-off group and the preoperative group (*p* = 0.003), the CI-on group and the preoperative group (*p* < 0.01), and the CI-on group and the CI-off group (*p* = 0.03) (see Table 4).

The preoperative average depression score was 7.5 ± 3.7, and the postoperative average anxiety scores were 5.9 ± 3.2 when the CI device was off and 5.3 ± 3.3 when the CI device was on. There were significant differences between the CI-off group and the preoperative group (*p* = 0.02) and between the CI-on group and the preoperative group (*p* = 0.002). There was no significant difference between the CI-on group and the CI-off group (*p* = 0.33) (see Table 4).

### 3.6. The Degree of Hearing Improvement after CI Is Correlated with the Degree of Tinnitus Reduction when the CI Device Is On but Is Not Correlated with Anxiety or Depression

According to the results summarized in Table 5, the CAP scores were 1.3 ± 1.3 before CI and 4.3 ± 1.3 after CI. There was a significant difference (*p* < 0.01), meaning that hearing was significantly improved after CI. Pearson's relative statistic was used to test the relationship between the hearing improvement degree (CAP) after CI and the tinnitus reduction degree (THI). The degree of hearing improvement after CI was correlated with the degree of tinnitus reduction when the CI device was on (*p* < 0.01), but when the CI was off, there was no significant difference (*p* = 0.6). Thus, the better the hearing improvement was, the less severe the tinnitus. However, there was no relationship between the degree of hearing improvement and the degree of anxiety/depression reduction. For the SIR, there was no difference before and after surgery.

### 3.7. Other Relative Relationships

Pearson's relative statistic was also used to determine the relationship between the Categories of Auditory Performance (CAP) and Speech Intelligibility Rating (SIR), CAP and tinnitus level, CAP and depression/anxiety level, CAP and hearing aid wearing duration, and SIR and hearing aid wearing duration. There were no relationships between these potentially related factors.

## 4. Discussion

Patients with profound or severe bilateral deafness have difficulty communicating with others, and some of them suffer from tinnitus, anxiety, and depressive stress. Cochlear implantation is a common treatment for patients with bilateral sensorineural hearing loss, which not only improves hearing but also relieves tinnitus, anxiety, and depression stress [7, 8, 10–15, 22]. Recently, customized music therapy has been shown to be an effective treatment for relieving severity level of chronic tinnitus, but the effect for relieving anxiety and depress is not clear [8].

In this retrospective study, we investigated the influence of cochlear implants on patients with profound bilateral deafness. According to our results, 94% of the CI patients suffered from tinnitus before CI surgery, and 77.1% of them had bilateral tinnitus, which is consistent with previous reports ([7, 12, 22]). The proportion of patients with severe and catastrophic tinnitus before CI surgery was 45.1%. Meanwhile, the proportion of profound BD CI candidates with anxiety and depression was high; 50.9% of them suffered from anxiety before CI surgery, 52.9% of them suffered from depression before CI surgery, and 66.7% of the CI candidates suffered from depression and anxiety. Thus, these factors greatly affect the physical and mental health of bilateral profoundly or severely deaf patients. Therefore, we focused on tinnitus distress and psychological comorbidities in the study.

Regarding the mechanism of tinnitus, it is generally agreed that tinnitus is generated within the brain in response to a reduction in auditory nerve fiber input from the cochlea to the brain. However, deafferentation appears to be necessary, but not sufficient, to produce tinnitus [5, 24, 25]. Deafferentation can result in tinnitus from the cochlear nucleus (CN) accompanied by homeostatic and timing-dependent plasticity. Thus, the mechanism of tinnitus explains why most tinnitus patients suffer from varying degrees of unilateral or bilateral hearing loss and why many patients with hearing loss do not have tinnitus clinically.

In previous reports, an additional benefit of unilateral cochlear implantation was subjective reduction of tinnitus [7, 10, 12, 22]. Similarly, according to our results, the THI and VAS scores were reduced significantly after CI surgery when the CI device was on. It is also interesting that although 77.1% of CI candidates had bilateral tinnitus, one-sided CI could suppress both unilateral and bilateral tinnitus symptoms. This phenomenon could be explained by the auditory conduction pathway and contributes to the understanding of the lateralization of tinnitus. The CN is the first brain station that receives input from the cochlea and is believed to be the origin of tinnitus. After auditory afferent input to the cochlear nucleus, most of the input signal is transmitted to the contralateral inferior colliculus (IC), and some is transmitted to the ipsilateral IC [5]. Therefore, unilateral CI can reduce tinnitus on both sides (i.e., the contralateral and ipsilateral sides).

Although the reduction in tinnitus was more significant in the CI-on state, we still observed that tinnitus distress was suppressed even when the CI device was off. This is a very interesting phenomenon that provides some clues to the mechanism of tinnitus treatment. Restored deafferentation could explain the reduction in tinnitus in the CI-on state. Auditory input by a CI device could offset deafferentation immediately and reduce tinnitus. After the CI device works repeatedly for a period of time, this counteraction might slowly remodel the auditory center. Therefore, some people may be able to partially compensate, and the plasticity in the auditory center is in a state of homeostasis when even the CI device is turned off. For CI patients with chronic tinnitus that has lasted for decades, their tinnitus could decrease immediately when the CI is on, which gives us great inspiration that we can treat tinnitus even if it has be present for a long time. In our study, 3 BD patients had no tinnitus before CI but had slight or moderate tinnitus after CI, which is also consistent with previous reports that CI could induce tinnitus [12]. For these patients, restored deafferentation may induce remodeled plasticity in the auditory center and then tinnitus. Overall, most CI candidates experience a reduced burden of tinnitus.

In this study, we also investigated the influence of CI on the psychological comorbidities of BD patients. According to our results, 73.1% of patients with anxiety showed suppression of symptoms after CI surgery, and 63% of patients with depression showed suppression of symptoms after CI surgery. It is obvious that CI can suppress anxiety and depression levels. One reason for these changes is that the patients can hear sound and can speak more fluently than before CI surgery; another reason might be that the tinnitus distress is decreased. According to our results, there is a positive correlation between the severity level of anxiety/depression and tinnitus before CI surgery. The postoperative situation is more complicated. There was a positive correlation between the severity level of depression and tinnitus, but there was no correlation between the severity level of anxiety and tinnitus (*p* = 0.06). Tinnitus and anxiety or depression are comorbidities that influence each other. This provided us an important point in the clinical treatment of tinnitus, as we need to treat tinnitus and symptoms of anxiety or depression, including insomnia, at the same time.

In addition, we also explored the relationship between the degree of hearing improvement (CAP) and the degree of tinnitus reduction. Hearing was significantly improved after CI, and the better the hearing improvement was, the less severe the tinnitus. Thus, better restoration of auditory deafferentation could reduce tinnitus severity.

For the SIR, there was no difference before and after CI surgery, which may be because these patients were postlingually deafened. In addition, we also explored the relationships between SIR and tinnitus severity, SIR and depression/anxiety severity, CAP and hearing aid wearing duration, and SIR and hearing aid wearing duration. There were no relationships between these potentially related factors, which may be due to the limited number of candidates included in this study.

## 5. Conclusion

There is a high prevalence of patients with bilateral profound or severe deafness suffering from severe tinnitus, anxiety, and depressive stress, which seriously affect quality of life. Cochlear implantation is an effective treatment not only for improving hearing but also for relieving tinnitus, anxiety, and depressive distress. Cochlear implantation could reduce unilateral and bilateral tinnitus more obviously with the CI on than off. For some patients, even if the CI device is off, tinnitus distress could be reduced. There was a significantly positive correlation between tinnitus severity and anxiety/depression severity. Although CI could induce tinnitus for some CI candidates without tinnitus, most CI candidates experienced a reduced burden of tinnitus. Hearing was significantly improved after CI, and the better the hearing improvement was, the less severe the tinnitus. The repair of deafferentation with cochlear implantation might be responsible for the improvement of all the above symptoms in addition to hearing loss, which is important for future clinical and research work.

## Figures and Tables

**Table 1:** Preoperative characteristics of CI candidates.

Preoperative characteristics	
Characteristics	Sum *n* = 51
Male : female, No.	24 : 27
Age at CI	41.0 ± 17.0 (19-74)
Hearing aid use proportion before CI (%)	60.8% (31/51)
Mean duration of severe deafness before CI (years)	8.0 ± 7.2 (0.5-27)
Tinnitus proportion before CI (%)	94.1% (48/51)
Catastrophic (78–100)	22.9% (11/48)
Severe (58–76)	25% (12/48)
Moderate (38–56)	20.8% (10/48)
Mild (18–36)	29.2% (14/48)
Light (0–16)	2% (1/48)
Anxiety proportion before CI (*A* ≥ 8)	51% (26/51)
Depression proportion before CI (*D* ≥ 8)	52.9% (27/51)
Anxiety or depression proportion before CI (*D* ≥ 8)	66.6% (34/51)

**Table 2:** 

Relationships between depression/anxiety level and tinnitus level					
Pre-CI	THI	*A*	Post-CI	THI	*A*
Pearson relative	1	0.576^∗∗^	Pearson relative	1	0.264
*p*	0.001^∗∗^		*p*	0.061	
Pre-CI	THI	*D*	Post-CI	THI	*D*
Pearson relative	1	0.622^∗∗^	Pearson relative	1	0.377^∗∗^
*p*	0.001^∗∗^		*p*	0.006^∗∗^	

^∗∗^Significant correlation at the 0.01 level (bilateral).

**Table 3:** 

THI and VAS scores before and after CI				
Type (%)	Pre-CI (A) (%)	Post-CI off (B) (%)	Post-CI on (C) (%)	*p* ^∗^ *p* ^∗∗^
Overall THI	53.1 ± 27.5	43.2 ± 26.2	29.1 ± 21.3	A&B *p* = 0.06
	94% (48/51)	98% (50/51)	94% (48/51)	B&C *p* < 0.01^∗∗^
				A&C *p* < 0.01^∗∗^

Catastrophic (78–100)	90.4 ± 7.1	66.7 ± 31.4	39.8 ± 32.2	A&B *p* = 0.02^∗^
	22.9% (11/48)	12% (6/50)	4.2% (2/48)	B&C *p* = 0.06
				A&C *p* < 0.01^∗∗^

Severe (58–76)	67.7 ± 6.1	54.8 ± 21.1	35.8 ± 20.1	A&B *p* = 0.06
	25% (12/48)	24% (12/50)	12.5% (6/48)	B&C *p* = 0.03^∗^
				A&C *p* < 0.01^∗∗^

Moderate (38–56)	48.4 ± 6.1	42.4 ± 8.1	32.6 ± 12.9	A&B *p* = 0.08
	20.8% (10/48)	20% (10/50)	14.6% (7/48)	B&C *p* = 0.056
				A&C *p* < 0.01^∗∗^

Mild (18-36)	29.0 ± 5.0	18.6 ± 9.1	13.8 ± 8.4	A&B *p* < 0.01^∗∗^
	29.2% (14/48)	22% (11/50)	35.4% (17/48)	B&C *p* = 0.1 A&C *p* < 0.01^∗∗^

Light (0-16)	12	32 ± 38.2	26 ± 10.7	A&B *p* = 0.03^∗^
	2% (1/48)	22% (11/50)	33.3% (16/48)	B&C *p* = 0.62
				A&C *p* < 0.01^∗∗^

VAS	3.9 ± 3.1	2.6 ± 2.8	1.7 ± 2.2	A&B *p* = 0.06
				B&C *p* = 0.02^∗^
				A&C *p* < 0.01^∗∗^

**Table 4:** 

Anxiety and depression scores before and after CI				
Score	Pre-CI (A)	Post-CI off (B)	Post-CI on (C)	*p* ^∗^ *p* ^∗∗^
Anxiety score	7.2 ± 2.5	5.6 ± 2.6	4.5 ± 2.4	A&B *p* < 0.01^∗∗^
				B&C *p* = 0.03^∗^
				A&C *p* < 0.01^∗∗^

Depression score	7.5 ± 3.7	5.9 ± 3.2	5.3 ± 3.3	A&B *p* = 0.03^∗^
				B&C *p* = 0.33
				A&C *p* < 0.01^∗∗^

*A*: anxiety score; *D*: depression score.

**(a):** 

CAP and SIR scores before and after CI			
Score	Pre-CI	Post-CI on	*p* ^∗^, *p*^∗∗^
CAP	1.3 ± 1.3	4.3 ± 1.3	*p* < 0.01^∗∗^
SIR	4.4 ± 0.8	4.5 ± 0.7	*p* = 0.6

**(b):** 

Correlation between CAP improve degree and tinnitus reduce degree			
CAP	THI	Pearson relative *r*	*p*
(Post- CI -P re-CI)	(Post-CI on- P re-CI)	0.387	*p* = 0.093^∗∗^
(Post- CI -P re-CI)	(Post-CI off - P re-CI)	0.011	*p* = 0.939

^∗∗^Significant correlation at the 0.01 level.

## Data Availability

The data that support the findings of this study are available on request from the corresponding author. The data are not publicly available due to privacy or ethical restrictions.
